# The challenges of establishing drowning registration system in Iran

**Published:** 2019-07

**Authors:** Enayatollah Homaie Rad, Hamid Heidari, Naeema Khodadadi Hassan-Kiadeh, Ali Davoudi-Kiakalayeh, Zahra Mohtasham-Amiri, Leila Kouchakinezhad-Eramsadati

**Affiliations:** ^*a*^Guilan Road Trauma Research Center, Guilan University of Medical Sciences, Rasht, Iran.

## Abstract

**Background:**

Adopting effective drowning prevention measures are dependent on collecting data on drowning rate, since drowning prevention requires the planning and policy-making on known risk factors. When interventions are carried out, data are still required to monitor and evaluate the evaluation of the strategy. Therefore, recording drowning cases accurately can be effective in its preventing. The purpose of this study was to provide the challenges of implementing a drowning registry.

**Methods:**

Various sessions were held with attendance of the experts from the related provincial organizations such as Red Crescent, EMS, Lifeguard, and Governor General Office, Ports and Shipping, among others, as well as the health experts from medical universities. The existing challenges were recognized using qualitative research and semi-structured interview method. All related challenges were mentioned and prepared through qualitative interviews with twenty experts. Finally, they were analyzed utilizing MAXQDA software.

**Results:**

The most important problems in implementing the drowning registration system included: 1- Lack of enough time to collect data on the registration system when providing emergency services on the beach 2- Lack of proper map and GPS system in identifying drowning locations 3- Lack of a single responsible organization for dealing with the beaches’ issues 4- Lack of correspondence in organization involved in drowning in northern coastal provinces 5- Lack of sufficient information about important variables related to the registration system 6- The absence of the rescue forces in some areas other than the sea protected areas and the lack of access to clinical data on drowning in these zones at night hours 7- Need for more cooperation by the Forensic Medicine Organizations of provinces to provide data

**Conclusions:**

The drowning registration system has many challenges that can mostly be solved by providing appropriate funding and right registering of drowning and purchasing appropriate equipment.

**Keywords:**

Drowning registration, Challenges, Coastal provinces

